# Nursing strategies for the mechanically ventilated patient

**DOI:** 10.3389/fvets.2023.1145758

**Published:** 2023-07-28

**Authors:** Cassandra Meitner, Rachel A. Feuerstein, Andrea M. Steele

**Affiliations:** ^1^Department of Small Animal Clinical Medicine, Small Animal Emergency and Critical Care, University of Tennessee College of Veterinary Medicine, University of Tennessee, Knoxville, TN, United States; ^2^Ontario Veterinary College, Health Sciences Centre, University of Guelph, Guelph, ON, Canada

**Keywords:** mechanical ventilation, nursing, veterinary technician, bundles, patient monitoring, standard of practice, VAP, nursing bundles

## Abstract

The goal of this manuscript is to provide a comprehensive and multi-disciplinary review of the best nursing practices of caring for mechanically ventilated patients. By reviewing human medicine literature, the authors will extrapolate procedures that have been found to be most effective in reducing the risk of mechanical ventilation (MV) complications. Paired with review of the current standards in veterinary medicine, the authors will compile the best practice information on mechanically ventilated patient care, which will serve as a detailed resource for the veterinary nursing staff. Written from a nursing standpoint, this manuscript aims to consolidate the nursing assessment of a mechanically ventilated patient, addressing both systemic and physical changes that may be encountered during hospitalization. The goal of this review article is to present information that encourages a proactive approach to nursing care by focusing on understanding the effects of polypharmacy, hemodynamic changes associated with MV, complications of recumbent patient care, and sources of hospital acquired infections. When applied in conjunction with the more technical aspects of MV, this manuscript will allow veterinary technicians involved in these cases to understand the dynamic challenges that mechanically ventilated patients present, provide guidance to mitigate risk, address issues quickly and effectively, and create an up-to date standard of practice that can be implemented.

## 1. Introduction

The mechanically ventilated veterinary patient requires significant nursing care and team collaboration for the best outcome. It is imperative that veterinary technicians understand the nursing care required (1). Understanding the rationale and purpose of these interventions will aid in ensuring compliance (1).

There are many chapters and books written on each of the subsequent topics. It is not the intention of the authors to go into detail about each aspect of care discussed, but rather summarize the most important points, and highlight how to address them. The authors encourage the reader to seek out additional resources for a more in-depth review of what is being discussed within this manuscript.

## 2. Preparation for mechanical ventilation

With any invasive procedure, preparation is the key to a successful outcome. Preparation should include assessment of mechanical ventilation (MV) equipment and supplies, ensuring adequate inventory and organization; collection of bedside nursing supplies; and development of facility protocols and education of clinical and nursing staff to ensure familiarity and knowledge of the protocols (2).

### 2.1. Ventilation equipment and supplies

A critical care ventilator is a capital expense for a veterinary clinic, and like any investment, this machine requires upkeep to ensure it is ready for use when needed. A patient requiring MV cannot wait for ordering of supplies or coordinating repairs, it must be ready in the moment it is needed. An important aspect of being prepared for any patient requiring MV is to regularly review the number, function and sterility of consumable supplies such as single-use circuits, humidifier chambers, suction materials and end tidal CO_2_ (EtCO_2_) cuvettes (3). Products often can only be reprocessed a particular number of times before performance deteriorates, making it important to keep detailed informational logs on how many times an item has been used, and always have a backup on hand. Assigning an individual staff member to maintain the ventilator(s) is important (4). This person should oversee the care and maintenance of the machine as well as inventory of supplies for the clinic (4). Having a system to review, order, and organize this inventory will help to ensure proper quantities are kept (5). The use of calendars, checklists, and a system to ensure communication when items are used is ideal to preventing gaps in product availability (6). During COVID-19, ventilator consumables became increasingly scarce as products went on allocation, meaning they were diverted to human use. In addition, many backorders occurred in the supply chain, making it increasingly important to maintain inventory accuracy.

### 2.2. Bedside nursing supplies

It has been proposed that, by reducing the steps required to find all supplies necessary to perform an intervention, compliance may be increased (4, 7). This has been suggested as a factor in reducing central line associated blood stream infections (CLABSI), where a central line cart, with all supplies needed for placement and maintenance of central lines, was successful in limiting the need to go to eight separate locations to find supplies (8). Intensive care nurses noted increased compliance in placement using the new protocol when all supplies were readily available (8). While the authors could not isolate the specific role of the catheter cart in the overall reduction of CLABSI identified in this study, it was suggested that the development and use of the cart, and subsequently ensuring it was stocked at all times with necessary items, played a role (7, 8).

Similarly, having any supplies necessary for the care of mechanically ventilated patients at the patient’s bedside may have benefits in veterinary patients. The authors suggest having a cart specifically for ventilated patients that houses all the supplies necessary to support the facility’s bundled approach to care (Table 1).

**Table 1:** Suggested items for bedside ventilator cart.

Airway	Bag-Valve Mask for emergency ventilation Sterile endotracheal tubes Laryngoscope Tube ties ET cuff manometer Replacement tracheostomy tubes/liners Suction supplies -airway and oral separated
Anesthesia supplies	Additional sedation/induction drug(s) Emergency drug doses calculated
Patient hygiene	Oral Care Supplies Sponge forceps Sterile gauze squares Saline flush Kidney bowl or other container Saline +/− chlorhexidine per facility protocol Eye Lubricant Suctioning tools ie Yankauer^®^ tip Waterless shampoo or Baby wipes
Care giver supplies	PPE as required for patient (gowns, mask, gloves) Sterile Glove selection Hand Sanitizer
Nursing needs	Tape Catheter flush Supplies to replace IVC if needed; Various size catheters, T-port Non- sterile gauze Needles and syringes of various sizes Alcohol swaps for IV ports
Ventilator supplies	Additional supplies that may need to be swapped out urgently (will vary based on ventilator brand/type) CO_2_ cuvettes Flow sensors Inspiratory/expiratory valves Patient filters Sterile water for humidifier

### 2.3. Ventilator bundle

The use of care bundles has been a significant advancement in preventing hospital-acquired infections related to invasive devices or procedures in human medicine (8). A bundle is defined as an evidence-based set of treatment goals that, when used together, promote optimal outcomes (1, 9). Bundles have become standard of care in human medicine in an effort to reduce ventilator associated pneumonia (VAP), urinary tract infections, bloodstream infections, and surgical site infections (10–13). In 2005, the Institute for Healthcare Improvement (IHI) proposed a four-item ventilator associated pneumonia (VAP) bundle that was promoted to improve MV outcomes (14). The IHI revised the bundle in 2010 adding a fifth component (14). While a bundle is most often created and implemented at the individual facility by a group of expert stakeholders after reviewing the current evidence and factoring in their patient population, some bundles have been foisted on member hospitals by overseeing organizations; with resultant reductions in reimbursement for non-compliance or not meeting target numbers (14).

A bundle incorporates current guidelines and evidence to produce three to five interventions that, when completed diligently at the prescribed intervals in a standardized manner, are shown to reduce the risk of complications (9). Numerous studies have identified a positive improvement in outcome when nursing or treatment care becomes standardized (1, 15–18).

There is a lack of veterinary-specific evidence in many areas that would support the implementation and use of a veterinary-specific bundle. It may be more prudent to discuss “best practices” to be used in each veterinary facility, created by extrapolation from the human literature, until such evidence is available.

The most common interventions that are found in human VAP bundles include:

- Elevation of the head and thorax approximately 30° (14–16)
- Daily “sedation vacation” to assess readiness to wean (14, 16)
- Peptic or stress ulcer disease prophylaxis (14–16)
- Deep vein thrombosis prophylaxis (14–16)
- Oral care with or without chlorhexidine; with or without toothbrushing (14, 15)
- Hand hygiene prior to touching any ventilator tubing or patient mouth (14).

Elements of the bundle created will vary by location, type of facility and be reflective of the types of conditions and patient population requiring mechanical ventilation. It is important to note many studies have shown limited impact or success that cannot be attributed to the bundle alone (14–16, 19).

### 2.4. Developing a facility protocol/best practices

The Agency for Healthcare Research and Quality has created a program that can assist in the development of protocols that is easily transferable to the veterinary world (20). The program is the Comprehensive Unit-based Safety Program (CUSP), and resources are readily available to assist in the implementation at https://www.ahrq.gov/hai/cusp/index.html (20). CUSP is modeled on many tenants of patient safety and encourages team collaboration to create protocols, enforcement through education and engagement, an understanding of “just culture,” and reassessing the protocols on regular intervals to ensure they are still relevant (20). The use of the four “E’s” further guides the protocols from start to finish (20);

1. Engage: How will this make the world a better place?
2. Educate: How will we accomplish this?
3. Execute: What do I need to do?
4. Evaluate: How will we know we made a difference?

With any process or quality improvement initiative, education is key to a successful implementation (8). Hands-on training and assessment should be completed, ensuring all nursing staff, clinicians, and ancillary staff understand the protocol as it is relevant to them (8). Ideally, staff should complete an assessment prior to working on the procedure on their own (8).

## 3. Ventilated patient care

The veterinary technician is often responsible for monitoring mechanically ventilated patients. While instruments and monitors are helpful in watching trends and minute-to-minute changes, a knowledgeable veterinary technician is instrumental in overall patient assessment (21). Changes in patient parameters should be investigated, and the clinician should be notified promptly. Nursing care may be limited by patient size, responsiveness to stimulus, or other factors. Some patients may require additional nursing needs beyond what is listed below.

### 3.1. Hand hygiene

Hand hygiene has been identified as one of the single-most important steps to decrease the risk of a variety of hospital acquired infections (HAI) (22–28). Despite the evidence, hand hygiene compliance is still considered an issue (23–28). Glove use has been associated with reduced handwashing compliance and outbreaks of multidrug resistant bacteria (22, 29–32). Although the use of gloves is often considered an alternative to handwashing, it is imperative that hands be washed before and as soon as gloves are removed, as the hands become contaminated in the process of glove removal (33).

In veterinary medicine, hand hygiene compliance has been investigated in small animal practices (general and specialty) and equine hospitals (34–40). Overall, compliance for washing hands between patient has been reported to be 18.2–41.7% (34–36, 38, 40). Among credentialled and uncredentialled veterinary technicians and veterinary support staff, reasons for not washing hands frequently included being “too busy” (72.5%) or an “unpleasant feeling on hands” (24.7%) (38). In human medicine, lack of compliance has also been attributed to skin irritation from constant handwashing or use of irritating products (41).

It is important to consider that various hand hygiene regimens exist and are equally effective at reducing bacterial load on one’s hands (35, 36). Offering a variety of products including mild, unscented, and moisturizing soap and hand sanitizers at patient’s bedside and other key areas may improve compliance (22).

Mechanically ventilated patients are susceptible to VAP and device-related HAI such as catheter-associated urinary tract infections (CAUTI), CLABSI, and other catheter-related bloodstream infection (CRBSI) (27). Most mechanically ventilated veterinary patients are immunosuppressed, which increases risk of HAI (42). As such, appropriate hand hygiene is imperative. The use of examination gloves with any ventilated patient is recommended; however, glove use should not be used in lieu of appropriate hand hygiene (33).

### 3.2. Monitoring

Nursing care and daily monitoring of any patient may be performed using Kirby’s Rule of 20 (43). The patient parameters in the Kirby’s Rule of 20 checklist are summarized in Table 2 (43). Monitoring the mechanically ventilated patient is imperative to identifying changes in patient status and implementing early interventions (21). At minimum, an electrocardiograph (ECG), capnography (EtCO_2_), and pulse oximeter (SpO_2_) should be monitored on any mechanically ventilated patient (44) (Table 3). Using a multiparameter monitor, the veterinary technician can monitor ECG, EtCO_2_, SpO_2_, and invasive and/or oscillometric blood pressure (21). In human medicine, respiratory therapists often additionally monitor driving pressures, transpulmonary pressures, and pressure-volume loops to ensure appropriate positive end-expiratory pressure (PEEP) and prevent excess distending pressure (44). Auscultation is a valuable tool, and all veterinary technicians monitoring a mechanically ventilated patient should be comfortable identifying changes in lung sounds, diminished lung sounds, and changes in heart sounds or rhythm (21).

**Table 2:** Adapted from Kirby’s Rule of 20.

Fluid balance	Oncotic pull	Renal function	Wound healing and bandages
Blood pressure	Heart rate, rhythm, and contractility	Gastrointestinal motility and integrity	Drug dose and metabolism
Electrolyte and acid–base	Albumin	Nutrition	Pain control
Red blood cells and hemoglobin	Mentation	Glucose	Nursing care
Coagulation	Oxygenation and ventilation	Immune status and antibiotics	Tender loving care

Information for table from Small Animal Emergency and Critical Care textbook. Second Edition, Chapter 2.

**Table 3:** Patient parameters.

Parameter	Definition/recording	Troubleshooting: always note any changes in patients’ condition (RR, HR, alertness to see if contributing factor in reading
TPR	Standard Temperature, Pulse, and respiration rate	Provide heat support or cooling as indicated Monitor ECG for any changes in heart rate or rhythm. Confirm lead placement and connection to monitor Respiration quality and periodic auscultation
Blood Pressure (direct/indirect) S/D MAP	Blood pressure to be measured via indirect Doppler^®^ or invasive (arterial catheter) Consistent technique includes noting location and cuff size If invasive available, periodic indirect reading to understand how the compare	*Invasive* Inspect transducer to confirm placement and set up Check Patency of arterial line *Non-Invasive* Check Doppler^®^ (battery, crystal, connections) Palpate pulses to confirm they are present Change locations of reading
EtCO_2_	End-tidal carbon dioxide	Check for tube occlusion Check connections Check for excess dead space, moisture build Check arterial blood gas
SpO_2_	Pulse oximetry Flat probe (reflectance) vs clip probe (transmittance)	Reposition or relocate sensor Assess monitor for battery life/plug in Check arterial blood gas

#### 3.2.1. Cardiovascular status

##### 3.2.1.1. Electrocardiogram

Continuous ECG monitors heart rate and rhythm, which allows the veterinary technician to identify any arrhythmias that may develop (21). An audible pulse tone, in addition to alarms set to a high and low heart rate alert for the patient, provides an opportunity for changes to be identified immediately. Investigating changes in heart rate may include investigating medication dosing and assessing the level of pain, sedation, and anxiety of the patient (21). The patient should also be assessed for hemodynamic stability (45).

##### 3.2.1.2. Blood pressure

Arterial blood pressure can be monitored invasively *via* an arterial catheter or non-invasively *via* Doppler® or oscillometer (21, 46). Invasive blood pressure (IBP) monitoring is the gold standard and should be used for the most accurate blood pressure measurement (21, 46, 47). Continuous IBP can rapidly alert the veterinary technician to changes in blood pressure and is particularly important should the MV patient require accurate titration of vasoactive medications such as norepinephrine or dopamine (21, 46). Positive pressure ventilation is associated with increased intra-thoracic pressure during inspiration, and can lead to decreased venous return, cardiac output and secondary hypotension, (45, 48). The variation of the arterial pulse pressure seen over a MV breath cycle is called the pulse pressure variation (PPV) (49). In mechanically ventilated patients, PPV analysis can help identify hypovolemia and provide insight into a patient’s responsiveness to fluid therapy (49–51). It is generally accepted that a drop in systolic blood pressure greater than 10 mmHg during inspiration compared with expiration is indicative of fluid responsiveness (52).

In patients where an arterial catheter cannot be placed, the use of non-invasive blood pressure (NIBP) monitoring *via* Doppler® or oscillometer may be used as an alternative (21, 47). If NIBP is used, the cuff size and limb used should be recorded as NIBP may be influenced by differences in cuff size or limb position (46). If oscillometric blood pressure is continuously monitored, correlation with a Doppler^®^ reading should be periodically assessed (46).

Troubleshooting sudden changes in blood pressure should include checking other vital parameters to establish whether there are concurrent changes in heart rate, pulse pressure/quality, mucous membrane color and capillary refill time (46). Troubleshooting changes in the IBP waveform should include assessing patency of the arterial catheter and transducer system, re-zeroing the transducer to atmospheric pressure, and ensuring that the transducer is correctly positioned in relation to the level of the patient’s right atrium (46). Additional troubleshooting for NIBP measurement can include assessing and repositioning the blood pressure cuff or evaluating volume status via POCUS.

##### 3.2.1.3. Fluid balance

Intravascular volume status and hydration can be monitored through serial body weights, physical exams, percent dehydration assessments, thoracic point-of-care ultrasound (T-POCUS) including a left atrium (LA) to aortic root (Ao) ratio and a mushroom view to evaluate cardiac contractility and left ventricle volume status. Stroke volume variation, pulse pressure variation, and plethysmographic variability index may be useful in assessing fluid responsiveness in mechanically ventilated patients (44, 49) MV patients should be weighed at least once a day. Prior to transport for weight, the patient should have a period of pre-oxygenation at 100% in case of inadvertent extubation during transport. The veterinary technician should be prepared for potential adverse events during transport with reintubation supplies, suction and sedation (53).Positive pressure mechanical ventilation has been associated with sodium and water retention, as well as pulmonary edema, particularly when fluids and or catecholamines are required to maintain adequate hemodynamic support (54). The syndrome of inappropriate antidiuretic hormone (SIADH) has also been seen in association with pneumonia and MV (55–57). SIADH can be caused by central nervous system disorders, pulmonary disease, as an adverse effect of medications such as opioids, or as a result of positive pressure ventilation itself (56) These patients may look adequately hydrated or show signs of fluid retention (weight gain), decreased to minimal urine output (UOP) with an elevated urine specific gravity (USG) (56, 57). Treatment with low dose furosemide (0.1–0.5 mg/kg IV as needed) may be indicated (57). Monitoring total fluid volume administered and comparing it to total fluid volume produced is a helpful way to recognize if a patient’s fluids needs are being met. This is often referred to as monitoring ‘ins and outs’, and when assessed as part of the patient’s overall status, these measurements can offer insight into patient fluid needs (46, 58).

#### 3.2.2. Oxygenation and ventilation

##### 3.2.2.1. Respiratory rate

Respiratory rate and effort are important to monitor particularly if the patient is on a mode that allows spontaneous breathing (59). A critical care mechanical ventilator will provide some information about spontaneous breaths, such as tidal volume and peak pressures; however, any change in effort exerted from the patient should warrant investigation that may include auscultation of the chest for any wheezes, crackles, increased, or decreased lung sounds (45, 60). Performing a TPOCUS to look for fluid, B-lines, or pneumothorax may also be beneficial. Any changes noted may warrant performing thoracic radiographs (53).

##### 3.2.2.2. Pulse Oximetry

Despite its limitations, pulse oximetry is an important non-invasive tool for monitoring oxygenation in the mechanically ventilated patient (61). The gold standard for oxygenation monitoring is the partial pressure of arterial oxygen (PaO_2_) measured on arterial blood sample (45, 62). However, arterial blood gas (ABG) sampling is invasive, and the peripheral oxygen saturation (SpO_2_) can be monitored continuously, non-invasively, and with relative accuracy in hemodynamically stable states with a pulse oximeter (44). The sigmoidal oxyhemoglobin dissociation curve has historically described the relationship between the PaO_2_ and SpO_2_, though it can be altered through changes in pH, temperature, PCO_2_ or 2,3-diphosphyoglycerate (61) When patients are receiving supplemental oxygen, the sigmoidal nature of the oxyhemoglobin dissociation curve decreases accuracy in predicting PaO_2_ over 100, as the SpO_2_ can read at 100% with a PaO_2_ anywhere between 100 and 500 (61). Once past initial stabilization on the ventilator, typically a target of 95% SpO_2_ should be the goal during mechanical ventilation to mitigate the risks between both hypoxia and hyperoxia (2, 21, 44). There has been recent research in dogs describing the use of pulse oximetry as a surrogate for arterial oxygenation which found that, while mechanically ventilated dogs were more predictable, SpO_2_ is not a clinically suitable surrogate for PaO_2_, and ABG is still the ideal oxygenation assessment (61). It is nevertheless common practice to use both pulse oximetry and ABG to guide therapy in the veterinary patient as well as evaluate the correlation between PaO_2_ and SpO_2_ (21, 44, 61).

There are two main types of probes for pulse oximetry: reflectance and transmittance probes. Reflectance probes can be secured around the distal limb or tail base, and a spot reading can be performed in the femoral area (63). It is important to recognize that poor circulation or perfusion can influence SpO_2_ (11). The transmittance probe can be clipped to any mucous membrane such as the tongue or lip (63). Both types of probes should be repositioned periodically to avoid damage to the tissue, specifically ulceration of the mucous membranes (63). Low SpO_2_ troubleshooting includes checking the breathing system for blockage, or failure of oxygen delivery, then repositioning of the probe (45). If a solution is not found quickly, consider temporarily increasing FiO_2_ and checking the PaO_2_ with an ABG (45).

##### 3.2.2.3. Capnography

Capnography is a critical tool for any intubated patient (45). Veterinary technicians monitoring MV patients should be familiar with capnography waveform analysis, as the capnograph provides information on the quality of the breath and the circuit (59). In healthy lungs, EtCO_2_ is often 2–5 mmHg lower than PaCO_2_ due to mixing of the exhaled alveolar gas with dead space gas (64, 65). Larger discrepancies between PaCO_2_ and EtCO_2_ can be due to increased dead space from too long of an endotracheal tube, or circuit tubing, HME, pulmonary hypoperfusion associated with hypovolemia or increased physiologic dead space from diseased lungs (44, 64).

When an arterial blood gas is performed, the PaCO_2_ should be compared to the EtCO_2_ (45). Ventilator settings that can change the PaCO_2_ include respiratory rate, inspiratory to expiratory (I:E) ratio, tidal volume, and peak pressure (45).

##### 3.2.2.4. Arterial blood Gas

When initially stabilizing a patient on a mechanical ventilator, having frequent ABG’s often helps the clinician adjust the ventilator settings to optimize oxygenation and ventilation (2). In addition to continuous blood pressure monitoring, an arterial catheter can be used for ABG sampling (21, 53). Assessing oxygenation includes assessing the PaO_2_:FiO_2_ (P:F) ratio if the patient is receiving supplemental oxygen or the A-a (Alveolar-arterial) gradient if the patient is not receiving supplemental oxygen (62).

ABG evaluation is also beneficial for assessing both acid–base status and electrolytes of the patient (46). When monitoring PaO_2_ on mechanically ventilated patients, aiming for a PaO_2_ of 80 mmHg may decrease the risk of oxygen toxicity(66). If PaO_2_ is above 150 mmHg, the FiO_2_ should be decreased at the clinician’s discretion (45). While many patients may require initial FiO_2_ of 100%, oxygen should be decreased as tolerated to an FiO_2_ of <60% with the first 24 h to further reduce the risk of oxygen toxicity, with continued weaning to FiO_2_ of 30–40% or less prior to liberation from the ventilator (45). However, if any issues or concerns arise, temporarily increasing the FiO_2_ to 100% may be indicated until the oxygenation issue is resolved (21, 45).

When calculating the patient’s P:F ratio, it is important to note the patient’s current FiO_2_ when obtaining an arterial blood gas, as a P:F ratio of less than 300 is indicative of acute lung injury, and a P:F ratio of less than 200 is indicative of acute respiratory distress syndrome (67). A normal P:F ratio is >400 (68).

##### 3.2.2.5. Troubleshooting

If any complications with the ventilator or airway arise, the FiO_2_ may be increased to 100% temporarily until the issue can be resolved, at which point it can be weaned back down to previous levels, so long as the patient tolerates these changes (45). If there are any concerns about the ventilator not ventilating appropriately, disconnecting the patient from the ventilator and providing manual positive pressure ventilation *via* a bag-valve-mask will allow more manual assessment of the respiratory system (53). Troubleshooting techniques for low tidal volume or low-pressure alarms may include checking the endotracheal tube cuff for leaks or checking to make sure the airway is still patent and that there are no blockages or leaks (50). When using tracheostomy tubes, this includes making sure the tube is still in place. Suctioning the tracheal tube while looking for a mucus plug or increased secretions and/or changing out the tracheal tube for a new sterile one may be necessary (53). Increasing the FiO_2_ to 100% prior to changing out the tracheal tube should be standard practice (21). Most modern ventilators will have an O_2_ suction mode that increases oxygen automatically for 3 minutes, reducing the risk of forgetting to decrease oxygen after the procedure. Occlusion of the endotracheal tube may be seen as a loss or increase of EtCO_2_, a sudden decrease in tidal volume, an increase in peak inspiratory pressure, and/or a decrease in oxygen saturation (2, 50).

A pneumothorax may occur from positive pressure ventilation itself (60) or iatrogenically from procedures such as inadvertent bronchial placement of a nasogastric tube (69). A pneumothorax will be seen as sudden decrease in tidal volume, an increase in airway pressure, rapidly increasing EtCO_2_, and rapid desaturation (45). The risk of pneumothorax highlights the importance of setting appropriate alarms for the patient to ensure these changes are identified quickly. The patient will typically have decreased lung sounds on one side, pulsus paradoxus, or sudden tachycardia and hypotension (60). If time allows, a TPOCUS scan can be performed to look for an absent glide sign, or thoracic radiographs can be performed (45). If a pneumothorax develops, a chest tube will need to be placed, and the patient will need to be put on continuous suction, as the pneumothorax is unlikely to seal with positive pressure ventilation (60).

Incorrect MV settings, patient discomfort or level of sedation are a few of the causes that can lead to a patient “bucking the ventilator.” The reason for the patient-ventilator asynchrony should be identified quickly, as increased work in breathing can negatively impact patient outcome (2). Hypercapnia, hypoxemia and hyperthermia may develop if a patient’s respiratory rate or effort aren’t addressed in a timely manner (2).

#### 3.2.3. Temperature

Body temperature can be monitored either intermittently or continuously using a rectal probe connected to the multiparameter monitor. The veterinary technician should be monitoring trends and providing heat support or cooling as necessary (21, 70). Potential causes for an increase in temperature may include new infection (pyrexia), patient/ventilator dyssynchrony or worsening of the underlying disease process (66–68, 70–72). Hyperthermia has been associated with delayed weaning from ventilatory support in humans (71, 73). It is important to identify the possible cause for the increased temperature, as hyperthermia and pyrexia may require a different treatment approach (21). Rechecking radiographs, white blood cell counts, or c-reactive protein concentrations may help to differentiate pyrexia from hyperthermia (74). Treatment of hyperthermia involves active cooling measures adjustment of sedation, and/or ventilator mode & settings (2). As MV patients are at higher risk of developing nosocomial infections, treatment of pyrexia may involve investigation into sources of infection (59). Potential causes of hypothermia may include over-sedation or decreased body fat, particularly in cats, small dogs, and pediatric patients (21).

#### 3.2.4. Mentation

Mentation, as well as pain and sedation level, should also be monitored. For heavily sedated patients, depth of anestshesia should be monitored, and patients should be kept on the least amount of sedation necessary (53). For patients with other comorbidities that do not require heavy sedation or full anesthesia to maintain adequate MV through a tracheostomy tube, monitoring mentation in the form of the modified Glasgow Coma Score (MGCS) may be helpful to track trends (75). For patients who can be ventilated while more alert, monitoring any changes in mentation can be an indication of worsening or improving prognosis (53).

Developing a standard sedated patient assessment can help to monitor trends and achieve optimal sedation level. The goal is to provide adequate MV by keeping the patient comfortable and calm with minimal sedation to allow for early mobilization as feasible (53). In humans, both undersedation and oversedation have negative effects on both patients and hospital staff, so frequent targeted analgosedative assessments are recommended to minimize these risks (76). These assessments include the use of pain scores and various sedation assessments and scales, which combine physical parameters with behaviors such as: respiratory drive, response to ventilation, coughing, tolerance to care, consolability, muscle tone, and facial expression (76). Most human assessments possess limitations or are unreliable, as they use neuromuscular blockade agents (76). Neurologically intact veterinary patients frequently require higher levels of sedation/anesthesia to tolerate MV (53). Veterinary patients who have a tracheostomy tube may require less sedation (53), and for these patients, the use of veterinary-specific sedation assessments could be very beneficial. There is work to create reliable sedation assessments in dogs: the SIESTA (SEAAV Integrated evaluation sedation tool for anesthesia) project is working to modify the Delphi method of sedation assessment for canines (77), and another research group has validated a canine sedation assessment (78). In human medicine, nursing directed sedation and mechanical ventilator weaning protocols have proven to be quite successful (79). With the help of sedation assessments for canine patients, a similar veterinary technician-directed weaning protocol might be possible for our veterinary patients.

### 3.3. Sedated patient considerations

Sedation protocols are initiated on a patient-by-patient basis, with the goal of promptly treating pain, providing adequate sedation for anxiety, and reducing cardiovascular adverse effects (21). In human medicine, nurses underestimate patients’ pain, despite a large percentage of intensive care patients reporting experiencing pain (54). There are limitations to the direct application of specific components of care, such as communication with patients for assessing pain or sedation needs, physical restrictions for positioning and initiation of physical therapy, however the insight provided is crucial to reflect on how we can better meet the needs of our patients. Veterinary patients are unable to describe the pain they feel, therefore, veterinary technicians should be even more diligent about looking for signs and symptoms of pain in intensive care patients. Modifications should be made based on the patient’s needs, co-morbidities, and response to therapy or stimulation (21, 53, 70, 79). A multi-modal approach to sedation will likely decrease the dose of each individual medication needed to achieve the appropriate level of sedation (21, 79–81).

Neuromuscular blockade may also be necessary to prevent patient ventilator dyssynchrony, reduce barotrauma, decrease oxygen consumption with severe respiratory failure, and control intracranial pressure or intraabdominal pressure spikes in patients sensitive to the medications included in specific sedation protocols (53, 82).

In the authors’ experience, other medications often prescribed by the attending clinician may include the prophylactic or therapeutic use of prokinetics, GI protectants, and antibiotics. If antibiotics are needed, the need and intensity of the antibiotic use should be evaluated frequently and tailored to the patient through use of culture and sensitivity when available (83). It is not our role as veterinary technicians to prescribe these medications, however, it is important that we understand their effects on the patient and any synergistic or antagonistic effects that may occur when using these medications simultaneously. Not only do we have to consider how the medications work together, but we should also be aware of the compatibilities of the medications we are using. There are many polypharmacy considerations, and often, limited IV access requires attention to drug compatibilities and possible interactions (84). Incompatibilities among the drugs that are used can lead to the formation of precipitate in the line, phlebitis at the catheter site, and inactivation due to pH changes (84).

Strategies to limit the mixture of incompatible drugs have been identified and are simple to implement in practice. If multiple ports are available, grouping medications to specific ports (designated analgesia and sedation, antibiotic, and intermittent infusion) can reduce a mixture of incompatible drugs, unintentional bolusing, or cessation of administration (85). It is important to know which drugs should not be stopped for any amount of time (norepinephrine or propofol) and which drugs can be held for a set period to time to allow for separate administration. In a study conducted by Négrier et al., providing a drug compatibility chart to reference patient-specific medications reduced the mixing of incompatible drugs by 10% (86). For medications where data is not available, there is evidence that adding an in-line filter is effective in reducing thrombi, sepsis, and renal or pulmonary complications (86). Although there is no evidence that all particulate matter can be removed, and there is currently no consensus on what size to use, there is evidence that filter size of 0.2–1.2 microns is more effective than the 5-micron filters (87, 88).

The main complications seen with MV include hemodynamic instability due to positive pressure ventilation, infections (ventilator associated and/or hospital-acquired), pneumothorax, ventilator-induced lung injury, medication side-effects, and the inability to discontinue ventilatory support (60). Other complications may include skin disturbances from prolonged recumbency or edema, increased gastric residual volume, and venous or arterial catheter complications. If the patient needs to be mechanically ventilated for a period longer than 3 days, a discussion about performing a tracheostomy may arise, in which case the nursing staff can prepare all the tracheostomy placement and care equipment (89). MV with a tracheostomy may have less complications in veterinary patients with underlying neuromuscular disease as opposed to underlying pulmonary disease, as mobile veterinary patients have a higher risk of inadvertent circuit disconnect (89).

The main complications seen with long term MV in dogs seems to include pneumothorax, oral and corneal ulceration, gastric distension, occlusion of tracheal tube, urinary tract infection, edema, and non-pneumonia related hyperthermia (90, 91). Patients receiving MV are often receiving a significant level of sedation or anesthesia. These medications affect the body’s natural reflexes, putting them at a higher risk for things like aspiration, keratopathy, and pressure sores (70).

An important part of nursing care for the mechanically ventilated patient is “tender loving care” (TLC) (70, 80). MV patients should receive as little stimulation as possible, and this can be facilitated by covering the eyes, placing ear plugs in ears, or consolidating major treatments in the same hour to allow longer periods of rest in-between (80). These treatments may be adjusted on patient needs or length of ventilation. Excessive noise pollution has been shown to negatively affect patients’ immune response, disrupt sleep patterns, and cause enough stimulation to require increased sedation (92). Other forms of TLC can include talking to the patient or having their owners visit for periods of time or having soft music playing next to the patient (70, 80). In humans, music has been shown to decrease anxiety and reduce sedation frequency (92).

### 3.4. Spontaneous awakening trial and assessing readiness to wean

In human medicine, patients can have sedation reduced or reversed, even while orally intubated (80). This can allow the medical team to explain to the patient what they are doing and ask for the patient’s input and participation as medical decisions are made with respect to weaning from mechanical ventilation. The daily assessment has a goal of trying to decrease the duration of MV for the patient when possible (80, 89). Decreasing the number of ventilator days has been associated with a decreased risk of VAP (93).

Readiness to wean and weaning criteria should be discussed daily (80, 89). Discussions may be around trying different ventilatory modes to challenge the patient for a period and build their respiratory strength in anticipation of weaning (89). There may be a decision to change the anesthetic protocol to one that is shorter acting (21, 89). Perhaps a plan is to have high flow nasal oxygen ready for when the patient is removed from the ventilator (94).

Oftentimes, it is clear that the patient is not ready to wean, however, there may be other benefits to a spontaneous breathing trial or SBT. Each day the team should review the patient’s readiness to wean and ensure all decisions are well-documented (89).

The more the entire care team communicates and understands the shared goals, the more likely it is that the entire team will invest themselves in the goals. With mechanically ventilated patients being among the most challenging cases for any veterinary team, it is imperative that each person on the team knows their role and understands the purpose behind what they are trying to accomplish.

While we can add anything into a bundle or protocol, empowering everyone to follow that protocol is paramount and more likely to lead to a successful outcome (1, 95).

### 3.5. Maintenance of endotracheal tube cuff pressure

Maintenance of endotracheal tube cuff pressure is another important piece of many ventilator bundles. Inadequate cuff pressure can increase the risk of VAP, however, overinflation of the endotracheal tube can lead to tissue necrosis (21). If a high-volume, low-pressure (HVLP) endotracheal tube is used (typically used with smaller endotracheal tubes), the cuff pressure can be measured every 4 hours and does not need to be deflated and repositioned (21). However, if a low-volume, high-pressure (LVHP) endotracheal tube is used, cuff pressure is not a reliable indication of adequate seal of the airway, and overinflation can lead to tracheal necrosis (96). For LVHP tubes, the cuff should be deflated and repositioned every 4 hours after oral suctioning (21). HVLP cuff pressure in dogs has been suggested to be between 18 and 25 mmHg (97). In cats, there are no published guidelines other than one study claiming 20–30 mmHg cuff pressure, or “minimal occlusion pressure” (98). Regardless, the minimum pressure that results in a seal (no leak on the ventilator) should be measured using a manometer and checked repeatedly, as anesthesia level and patient positioning changes to ensure it remains adequate (21). The cuff can be inflated to the optimal range using a cuff manometer, thus mitigating the risk of either aspiration as a consequence of inadequate inflation, or pressure necrosis associated with overinflation (99). Daily endotracheal tube replacement is no longer recommended. Endotracheal tube replacement should be considered only when increased resistance is observed on the ventilator waveforms and secretion build-up cannot be resolved by suctioning.

Repositioning the ties that secure the tube should be done every 4 h to reduce the risk of tissue damage of the lips (21). Ties should be replaced every 24 h, or when a new tracheal tube is placed (21).

### 3.6. Elevation of the head and thorax

A 30–45° elevation of the head of the bed for mechanically ventilated patients has been a standard intervention in VAP bundles since their first use in 2005 (17). The elevation of the head and thorax has also been reported as one of the more complied with strategies in the ventilator bundle (15, 18). A body position of >30-degree incline can help reduce the work of breathing (100). Elevation of the head and thorax also helps avoid aspiration of refluxed gastric contents and oropharyngeal secretions (17).

While not examined in veterinary patients, it is the authors’ experience that elevation of the head of the bed is easily accomplished with foam wedges, tables, cribs with “reverse Trendelenburg” tops, or blankets and pillows. More research is required to see if veterinary patients will experience the same benefits as seen in humans, however, it is unlikely that elevating the head and thorax would be harmful (21).

### 3.7. Recumbency

Patients who are mechanically ventilated for an extended period are affected by many physical negative factors (101). Polyneuropathy and generalized weakness from immobility are among the main things experienced, secondary to side effects from the medications used for sedation/anesthesia (107). Additional injury may include pressure sores, dermal burns from prolonged heat support, fecal or urinary scald, and peripheral edema (70). Passive range of motion can help with active muscle fibers and reduce joint stiffness (70, 101). Changing body positions will improve ventilation, promote lung secretion clearance, increase oxygenation, and increase lung volume (53, 101). Rotating patients on a regular basis can help reduce the pressure and reduce the risk of ulcer formation over specific risk zones including: the skin overlying the scapulohumeral articulation, the greater trochanter and the thirteenth rib (107). Using a pressure relieving mat will also be beneficial to the overall comfort of the patient (107). If heat support is being used, it is important to check body temperature on a regular basis and use only what is needed for support (70). Mechanically ventilated patients are at higher risk for thermal burns, as they cannot move away from heat (70). The veterinary technician has an important role in ensuring patient comfort by changing the patient’s position frequently, adding padding to boney areas to reduce the risk of pressure sores, and making sure the bedding is clean and dry (70).

### 3.8. Oral care

Oral care is used to reduce the bacterial load in the mouth, and thus reduce the risk of contaminated oral secretions entering the trachea around the endotracheal tube or when orally intubating a patient (21, 103). Oral care can also keep the tissues of the mouth moist (103). Hand hygiene when handling the patient’s mouth, endotracheal tube, adapters, and circuit is important to prevent further colonizing the mouth (21). Despite oral care of the ventilated patient being identified as a primary mechanism for reducing the risk of VAP (104), there are no guidelines currently for proper oral care in ventilated dogs and cats. Standardized oral care has been implemented in human medicine through the use of prepackaged kits designed to be used over 24 h or facility created kits with all necessary supplies (104). While commercial prepackaged kits are likely not applicable to a veterinary facility, creating oral care kits in an effort to standardize oral care and compliance could be beneficial (104). As with many other procedures, ensuring all staff understand the necessary steps through education and training and protocol development is very important (18, 103, 105).

Oral care protocols should include proper oropharyngeal suction technique and mechanical cleaning of all surfaces within the mouth, usually on 4–8 h intervals (21). Mechanical cleaning can be accomplished by using a sterile instrument, such as a sponge forcep, to hold gauze squares soaked in saline while wiping all surfaces of the mouth and teeth (103). Caution should be used to avoid trauma to the teeth or oral mucosa, and it is very important that the veterinary technician note any oral lesions (21). The use of chlorhexidine at many varying concentrations as a mouthwash in veterinary patients has not been studied for efficacy, and anecdotally, may contribute to oral irritation and ulceration, as has been found in some human patients (106, 107). The use of sterile saline alone versus chemical antiseptics such as chlorhexidine, povidone iodine, and triclosan has shown to have similar reduction of risk of VAP (103) and is likely sufficient in the veterinary patient until further research is conducted on the use of other antiseptics in that patient population. Oropharyngeal suctioning can be accomplished with sterile oral suctioning implements (21). Specialized subglottic suction endotracheal tubes are available (108) but have not been studied in veterinary patients. Suctioning should be accomplished with a vented suction tube such as a Yankauer^®^ rigid suction vented tip or similar implement for good control while suctioning (108). The use of a clean laryngoscope can provide lighted visualization. Maintaining cleanliness of the implement between uses and replacing/reprocessing the suction tip every 24 h may be beneficial (15, 104).

### 3.9. Eye care

Sedation and anesthesia cause lagophthalmos, which can lead to keratopathy (109). In addition to that, many of our medications used for sedation or pain control can cause a decrease in tear production (110). The most common way to avoid this is to use a lubricant and flush alternating combination, but there has also been a success with a moisture chamber technique (goggles, or “doggles”) (111). Every 24 h, a fluorescein stain evaluation should be performed to monitor for development of ulceration. Patients receiving prolonged ventilation may benefit from a corneal culture and prophylactic topical antibiotic ointment (21, 53, 112).

### 3.10. Bladder care

Bladder management is an important detail when nursing a mechanically ventilated patient (70). A urinary catheter can be placed to help keep the patient clean and dry, can reduce the amount of manipulation needed for manual bladder expression, and can be a tool used to assess hydration status (70). Although beneficial, urinary catheters are not without risk. Catheter associated infections result in prolonged hospitalization and increased medical costs (113). Assessing the patients need, standardized placement protocol and care, and removing the catheter as soon as possible have been shown to reduce the risks of CAUTI (114). Veterinary technicians should investigate and report UOP. A patient on intravenous fluids should have a UOP of 1–2 mL/kg/h, if less than 1 mL/kg/h, the clinician should be notified (115). The patency of the urinary catheter should also be investigated, particularly if a sudden drop in UOP is noted (70).

Troubleshooting can involve checking the urinary catheter line to ensure no occlusion, ultrasound scanning of the urinary bladder to ensure the bladder is empty, and possibly aseptically flushing the urinary catheter line to ensure patency. The line should flush and aspirate easily (58). If a truly low UOP is discovered, checking a urine specific gravity may be helpful to the clinician in guiding any changes to the treatment plan. If a urinary catheter is not an option, then bladder assessment and manual expression may be necessary, and care should be taken to ensure the patient remains clean and dry to prevent urine scald (70). Changing bedding when necessary, using moisture wicking pads under the patient, and cleaning the patient with waterless shampoo or a bedside spot-bath are just a few of the ways to prevent scald (70).

### 3.11. Nutrition

Nutrition should be addressed early during MV as it is likely beneficial for recovery (116). Patients already face risks of weakened respiratory muscles developing while prolonged sedation and ventilation occur, and malnutrition can compound these effects (117). Nutrition can be administered parentally *via* central line or enterally. In some cases, parenteral (PN) is necessary, but there are many potential risks associated with its use (118). Enteral nutrition is the safer and preferred route in the majority of patients.

In veterinary medicine, a common route of EN is *via* nasogastric tube (119). This allows for monitoring of gastric residual volume (GRV) and may help identify the need for medication to enhance GI motility (116). It is common practice to routinely aspirate and measure GRV; however, there may be little evidence to support this as being beneficial to the patient (53). Reduction of GRV may decrease incidence of vomiting and regurgitation, however, lower GRV is not necessarily associated with a lower instance of VAP (116). A negative side effect of regular GRV measurement is decreased total caloric intake for the patient and changes in the electrolyte status (116). Establishing a care protocol for ventilator patients receiving enteral nutrition would include patient positioning, establishing a target GRV goal and diet selection (117). Aside from providing nutritional support, a nasogastric tube is beneficial for administration of medications that can help minimize the dosages needed for injectable opioids or sedation. Use of trazodone has been shown to help reduce the amount of needed propofol for ventilated patients (120). Consideration should be had to determine what each patient needs (121).

### 3.12. Medication, dosing

Despite limited published recommendations for anesthetic protocols in long term mechanical ventilation (122), there have been two protocols evaluated in dogs. One protocol consisted of diazepam (0.5 mg/kg/h) or midazolam (0.5 mg/kg/h), fentanyl (0.018 mg/kg/h), and propofol (2.5 mg/kg/h). A second protocol consisted of diazepam (0.5 mg/kg/h) or midazolam (0.5 mg/kg/h), morphine (0.6 mg/kg/h), and medetomidine (0.001 mg/kg/h) (81). In humans, dexmedetomidine is widely used in the management of mechanically ventilated patients and has been shown to reduce duration of mechanical ventilation while also reducing the risk of delirium in humans compared with propofol or midazolam (123), despite of the risks of hypotension and bradycardia (124) Other medications that may be considered as constant rate infusions to maintain adequate sedation/analgesia include propofol: 50–400 mcg/kg/min, diazepam (or midazolam): 0.1–0.5 mg/kg/h, dexmedetomidine: 0.5–3 mcg/kg/h, fentanyl: 2–8 mcg/kg/h, ketamine: 0.1–1 mg/kg/h, and butorphanol: 0.1–0.5 mg/kg/h (45).

### 3.13. Bloodwork

Serial bloodwork monitoring incorporates many of Kirby’s Rules via monitoring of albumin, electrolyte and acid base, red blood cell (RBC), hemoglobin, and glucose (43). Daily lab work should include a minimum of packed cell volume (PCV), total solids (TS), blood glucose, lactate, and blood gas (either venous or arterial), and daily or alternate-day monitoring of complete blood count (CBC), chemistry and electrolytes (43, 65). If a coagulopathy is suspected due to a decrease in PCV, coagulation monitoring might include checking prothrombin time (PT)/ activated partial thromboplastin time (aPTT) or viscoelastography (43). In addition to lab work, renal function can be monitored through serial evaluation of urine output and urine specific gravity (65).

### 3.14. Vascular access and maintenance

Vascular access can be challenging in critical patients for many reasons. Care of catheters is imperative for ongoing care and the safety of the patient (125). Recent studies have shown little evidence to support a scheduled replacement of peripheral intravenous catheters. Instead, it is recommended that a systematic evaluation of clinical issues and replacement when indicated occur (126). For central venous catheters, establishing a protocol for placement, handling, and maintenance, in addition to ensuring personnel are familiar with the protocols, are instrumental in reducing the risk of complications (127). For some patients, arterial catheters are placed for direct blood pressure monitoring and serial arterial blood gas sampling (46). Care for these catheters often-included scheduled flushing and evaluation looking for signs of loss of patency, phlebitis, pain, or bleeding (102). Depending on the disease process, some patients may have a variety of additional catheters, tubes, or drains to manage (70). Monitoring of the insertion site for development of swelling, redness or discharge, changes in placement seating, and bandage changes should be done as needed (126).

### 3.15. Record keeping

An important part of monitoring is detailed record keeping (70). In addition to a standard ICU/CCU treatment sheet which would involve hourly treatments, nursing care plan, and medications, mechanically ventilated patients may benefit from a designated ventilator flow sheet (53). The ventilator flow sheet should have an appropriate patient label, date, and page number. It should include information about the machine settings for patient maintenance, how the patient is responding to those settings, and basic patient monitoring and nursing care notes (Table 4). Recorded readings can be taken at any desired interval the clinician chooses. If the case is dynamic and requires many changes to the treatment plan, more frequent readings (5–15 min) may be indicated. If the case is more stable, less frequent readings (15–60 min) may be adequate (53). This will allow for a quick view of trends and early identification of problems so that, with clinician oversight, adjustments can be made to the treatment plan (53).

**Table 4:** Front page of suggested MV patient monitoring flowsheet: settings should be modified to match facility machine.

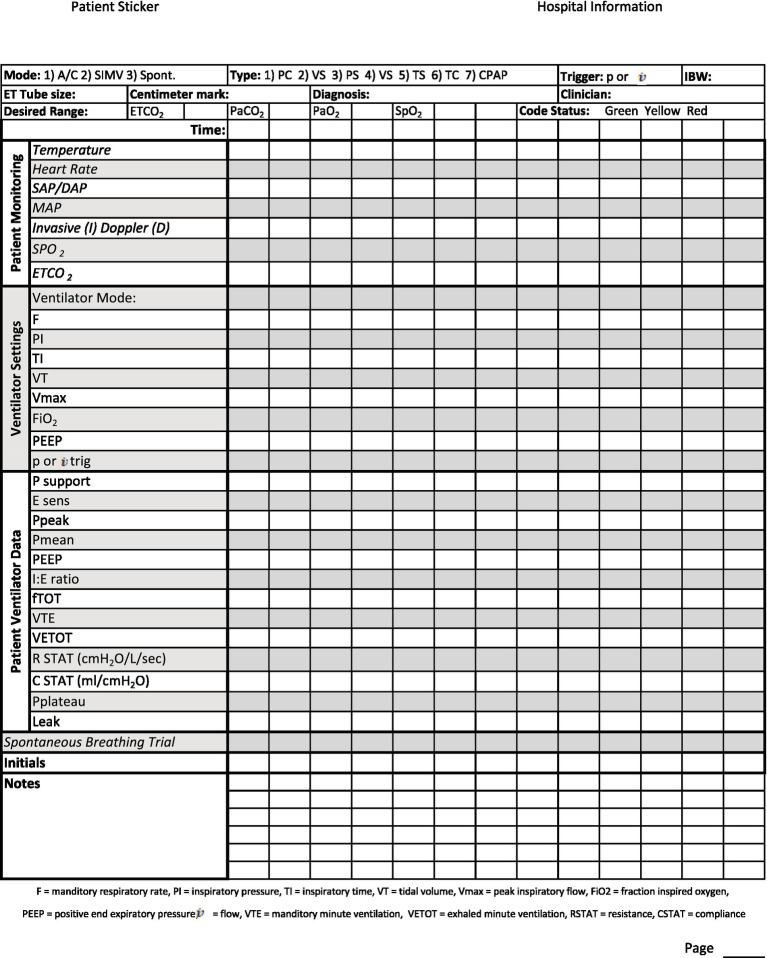
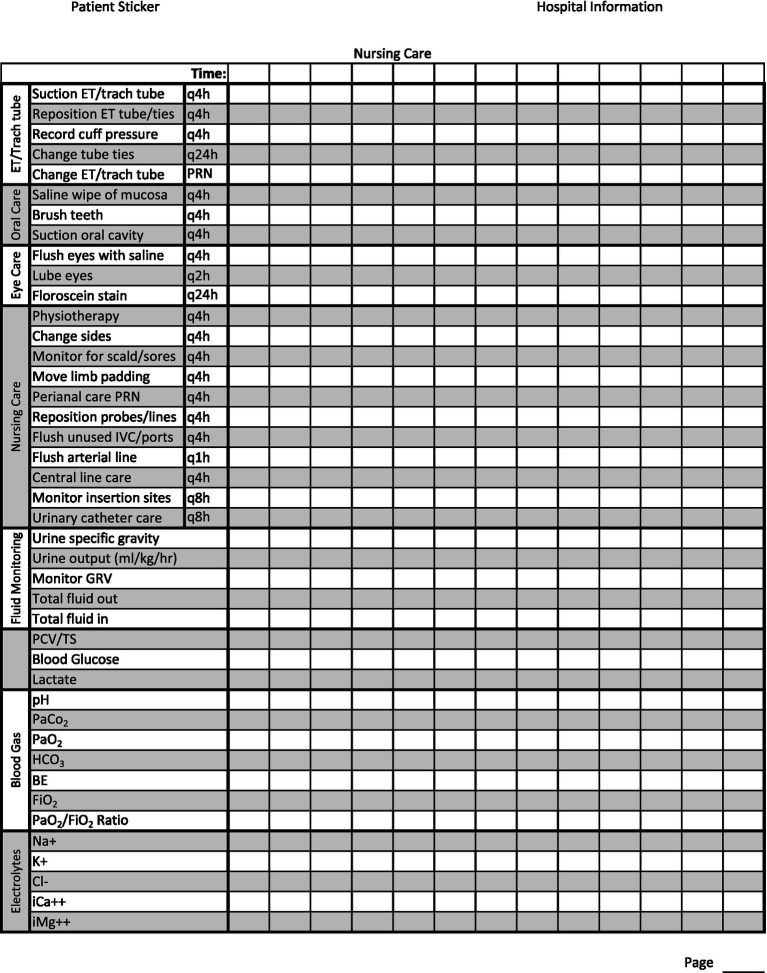

Back page of suggested MV patient monitoring flowsheet.

## 4. Discussion

It is imperative that we regularly reassess how we monitor and care for our critical veterinary patients based on what we learn from our human counterparts. A common goal in both human and veterinary literature is the need to reduce the risk of Ventilator Associated Events. The authors recognize that there is not always a direct conversion from human medicine to veterinary medicine, however some application of findings may prove to be equally advantageous to our veterinary patients.

MV patients pose a complex nursing opportunity, and it’s evident that more research is needed in veterinary medicine to specifically identify the benefits or detriments of certain aspects of care. Diligence and attention to ongoing changes in patient status, combined with detailed nursing care, will provide the best chance for a positive outcome for your patient.

## Author contributions

CM patient care, table creation, edit and organization introduction and conclusion. RF ventilation patient monitoring, edit and organization. AS ventilator machine research, bundles, patient care, formatting and input of references, and organization of content. All authors contributed to the article and approved the submitted version.

## Conflict of interest

The authors declare that the research was conducted in the absence of any commercial or financial relationships that could be construed as a potential conflict of interest.

## Publisher’s note

All claims expressed in this article are solely those of the authors and do not necessarily represent those of their affiliated organizations, or those of the publisher, the editors and the reviewers. Any product that may be evaluated in this article, or claim that may be made by its manufacturer, is not guaranteed or endorsed by the publisher.
